# RBM4a-SRSF3-MAP4K4 Splicing Cascade Constitutes a Molecular Mechanism for Regulating Brown Adipogenesis

**DOI:** 10.3390/ijms19092646

**Published:** 2018-09-06

**Authors:** Hui-Yu Peng, Yu-Chih Liang, Tse-Hua Tan, Huai-Chia Chuang, Ying-Ju Lin, Jung-Chun Lin

**Affiliations:** 1 School of Medical Laboratory Science and Biotechnology, College of Medical Science and Technology, Taipei Medical University, Taipei 110, Taiwan; m609104003@tmu.edu.tw (H.-Y.P.); ycliang@tmu.edu.tw (Y.-C.L.); 2 Ph.D. Program in Medicine Biotechnology, College of Medical Science and Technology, Taipei Medical University, Taipei 110, Taiwan; 3 Immunology Research Center, National Health Research Institutes, Zhunan 35053, Taiwan; ttan@nhri.org.tw (T.-H.T.); cinth@nhri.org.tw (H.-C.C.); 4 School of Chinese Medicine, China Medical University, Taichung 40402, Taiwan; yjlin@mail.cmu.edu.tw

**Keywords:** alternative splicing, brown adipocytes, MAP4K4, RBM4a, SRSF3

## Abstract

An increase in mitogen-activated protein kinase kinase kinase kinase 4 (MAP4K4) reportedly attenuates insulin-mediated signaling which participates in the development of brown adipose tissues (BATs). Nevertheless, the effect of MAP4K4 on brown adipogenesis remains largely uncharacterized. In this study, results of a transcriptome analysis (also referred as RNA-sequencing) showed differential expressions of MAP4K4 or SRSF3 transcripts isolated from distinct stages of embryonic BATs. The discriminative splicing profiles of MAP4K4 or SRSF3 were noted as well in brown adipocytes (BAs) with RNA-binding motif protein 4-knockout (*RBM4^−/−^*) compared to the wild-type counterparts. Moreover, the relatively high expressions of authentic SRSF3 transcripts encoding the splicing factor functioned as a novel regulator toward MAP4K4 splicing during brown adipogenesis. The presence of alternatively spliced MAP4K4 variants exerted differential effects on the phosphorylation of c-Jun N-terminal protein kinase (JNK) which was correlated with the differentiation or metabolic signature of BAs. Collectively, the RBM4-SRSF3-MAP4K4 splicing cascade constitutes a novel molecular mechanism in manipulating the development of BAs through related signaling pathways.

## 1. Introduction

Adipose tissues are known to preserve food-derived lipids as excess energy and function as an endocrine organ for energy homeostasis by secreting related hormones [1]. Dysfunction of the physiological roles of adipose tissues occurs with an expanded mass of white adipocytes (WAs) that subsequently induce human obesity [2]. In contrast, brown adipocytes (BAs) were discovered to execute non-shivering thermogenesis by dissipating stored lipid droplets in small rodents and infants [3]. Several studies revealed the impact of cold exposure and exercise on inducing white-to-brown shift within white adipose tissues (WATs) [4,5]. Therefore, BAs are considered a potential target for combating human obesity and related diseases [6]. Lineage tracing analyses indicated the existence of Myf5-positive progenitors for the development of myocytes and BAs [7]. Regulatory factors that participate in the differentiation of distinct adipocytes were uncovered using transcriptome analyses, but the post-transcriptional regulation involved in the developmental process of BAs is still largely being debated.

Alternative splicing (AS) functions as a post-transcriptional mechanism involved in expanding the diversity or manipulating the expression profiles of mammalian genomes [8]. Cellular process and corresponding function is specified by accurately executing AS events in a spatiotemporal manner, whereas imbalanced splicing regulation acts as a pathogenic cause toward cellular defects and diseases [9,10]. The interplay between trans-factors, such as the serine/arginine splicing factor (SRSF) and the heterogeneous ribonucleoprotein (hnRNP) protein, and corresponding elements constituting the regulatory mechanism involved in determining accurate splicing profiles [11,12]. Development of transcriptome analyses brought a comprehensive insight into tissue- and stage-specific AS profiles in the genome-wide era [13]. Results of transcriptome analyses revealed several BA-related splicing events, such as insulin receptor and serine/arginine protein kinase 1 in our previous studies, which participated in manipulating differentiation and the physiological function of brown adipose tissues (BATs) [14,15,16].

Results of transcriptome analyses indicated a shift in splicing profiles of SRSF3 (previously referred to as SRp20) and mitogen-activated protein 4 kinase 4 (MAP4K4) during brown adipogenesis in this report. Moreover, altered splicing patterns of SRSF3 and MAP4K4 transcripts were noted in RNA-binding motif protein 4a (RBM4a)-depleted BATs. The sequence-specific mechanism that participates in programming splicing profiles of the SRSF3-MAP4K4 splicing cascade was further characterized. Temporal expressions of MAP4K4 variants exhibited discriminative impacts on expressions of BA-specific factors and the mitochondrial activity of BA-like cells through manipulating the activity of c-Jun N-terminal kinase (JNK) signaling. Those results disclosed the impacts of a novel splicing cascade involved in BA development.

## 2. Results

### 2.1. Transcriptome Analyses Reveal Differential Expressions and Splicing Profiles of MAP4K4 and SRSF3 in Differentiating and RBM4a^−/−^ BAs

In our previous study, results of transcriptome analyses disclosed discriminative splicing profiles of 186 genes from a total of 13,217 splicing events in postnatal RBM4a^−/−^ BATs compared to wild-type (WT) BATs [15]. Among those candidates, the splicing profiles of mitogen-activated protein kinase kinase kinase kinase 4 (MAP4K4) and SRSF3 transcripts were altered after ablation of endogenous RBM4a in postnatal BATs. As shown in Table 1, *MAP4K4* isoforms 3 and 4 are major transcripts expressed by postnatal BATs (Table 1A; fragments per kilobase of transcript per million mapped reads (FPKM) = 15.15 and 14.76, respectively), whereas *MAP4K4* isoforms (Iso) 1 and 3 were mainly transcribed in embryonic BATs (Table 1A; FPKM = 42.23 and 32.43, respectively). In contrast, depletion of RBM4a mediated a drastic shift in *MAP4K4* Iso 3 to Iso 2 (Table 1A, FPKM = 20.32) in embryonic BATs, and reduced expression of MAP4K4 Iso 4 was noted in postnatal *RBM4a^−/−^* BATs (Table 1A, FPKM = 5.64) compared to the WT counterparts. Relatively high expressions of total *MAP4K4* transcripts were observed in embryonic and postnatal WT BATs (Table 1A, lower, FPKM = 32.8794 and 71.2371, respectively) compared to *RBM4a^−/−^* BATs (Table 1A, lower, FPKM = 17.3019 and 30.2237, respectively). Moreover, results of transcriptome assays showed an increase in the relative level of the non-coding *SRSF3* transcript (NR_036613) in postnatal WT BATs compared to that of embryonic WT BATs (Table 1B, PII = 53.68% and 13.91%, respectively), whereas expressions of the coding *SRSF3* transcript (NM_013663) was elevated in embryonic and postnatal *RBM4a^−/−^* BATs (Table 1B, FPKM = 213.5991 and 54.33154, respectively) compared to those of the WT counterparts (Table 1B, FPKM = 108.2105 and 10.98038, respectively). These results disclosed the reprogrammed expression and splicing profiles of *MAP4K4* and *SRSF3* throughout brown adipogenesis, which is relevant to the abundance of RBM4a.

### 2.2. Splicing and Expression Profiles of MAP4K4 and SRSF3 Transcripts are Reprogrammed Throughout BAT Development

**Figure 1:**
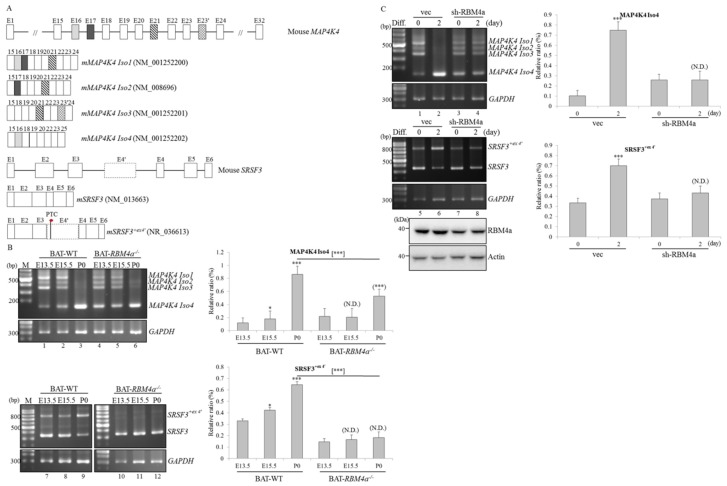
Splicing profiles of *mitogen-activated protein kinase kinase kinase kinase 4* (MAP4K4) and *serine/arginine-rich splicing factor 3* (SRSF3) transcripts are reprogrammed during brown adipogenesis. (**A**) The schemes respectively present the exon composition of mouse *MAP4K4* and *SRSF3* transcripts. (**B**) Total RNAs prepared from embryonic (E13.5 and E15.5) and postnatal (P0) brown adipose tissues dissected from wild-type (WT) and *RBM4a^−/−^* mice (*n* = 4) were subjected to RT-PCR assays using specific primer sets as listed in Supplementary Table S1. The bar graph presents relative levels of the *MAP4K4* Iso 4 and *SRSF3^+ex4^^′^* transcripts. (**C**) C3H1oT1/2 cells were respectively transfected with empty vector or the *RBM4a* targeting vector (sh-RBM4a), followed by culturing in growth medium and differentiating medium for 48 h. Total RNAs extracted from non-differentiating (0 day) and differentiating cells (2 day) were subjected to RT-PCR assays using indicated primer sets (*n* = 4). The bar graph shows relative levels of *MAP4K4* Iso 4 and *SRSF3^+ex4^^′^* transcripts. Signal densities of the RT-PCR products were quantified using TotalLab Quant Software. Quantitative results are shown as the mean ± SD. Statistical significance was determined using Student’s unpaired *t*-test (* *p* < 0.05; ** *p* < 0.01; *** *p* < 0.005), N.D., No Difference.

The reviewed sequences indicated that the *MAP4K4* gene generated four transcripts by alternatively selecting exons 16, 17, 21, and 24 (Figure 1A, upper). Retention of *SRSF3* intron 3 (also referred to as exon 4′) resulted in the generation of premature termination codon-containing transcripts which were considered to be candidates of the nonsense-mediated decay pathway (Figure 1A, lower) [16]. As shown in Figure 1B, results of the RT-PCR showed that the majority of *MAP4K4* transcripts were Iso 1 and 2 generated in embryonic (E)13.5 and E15.5 BATs (Figure 1B, lanes 1 and 2), whereas predominant expression of *MAP4K4* Iso 4 was noted in postnatal (P0) BATs isolated from WT mice (Figure 1B, lane 3). In contrast, postnatal *RBM4a^−/−^* BATs exhibited a relatively low level of *MAP4K4* Iso 4 (Figure 1B, lane 6, PII = 52.82%) compared to that of postnatal WT BATs (Figure 1B, lane 3, PII = 86.38%). RT-PCR results showed a gradual increase in relative levels of *SRSF3^+ex4′^* transcripts in postnatal WT BATs compared to those of embryonic BATs (Figure 1B, lanes 7–9). Nevertheless, splicing profiles of *SRSF3* continued to be sustained throughout the development of *RBM4a^−^*^/*−*^ BATs (Figure 1B, lanes 10–12). The reprogramming of *MAP4K4* and *SRSF3* transcripts was consistently represented using an in vitro cell model. As shown in Figure 1C, splicing profiles of the *MAP4K4* and *SRSF3* transcripts respectively shifted to *MAP4K4* Iso 4 and *SRSF3^+ex4′^* transcripts in the presence of the differentiating cocktail (Figure 1C, lanes 2 and 6) compared to those of C3H10T1/2 cells cultured in growth medium (Figure 1C, lanes 1 and 5). In contrast, splicing profiles of *MAP4K4* and *SRSF3* transcripts in *RBM4a*-depleted cells showed no response to the presence of the differentiating cocktail (Figure 1C, lanes 4 and 8) compared to those of non-differentiated cells (Figure 1C, lanes 3 and 7). These results suggested a potential link between RBM4a abundance and splicing profiles of the *MAP4K4* and *SRSF3* transcripts throughout brown adipogenesis.

### 2.3. RBM4a Exerts a Specific Influence on Modulating Splicing Profiles of MAP4K4 and SRSF3 Transcripts

**Figure 2:**
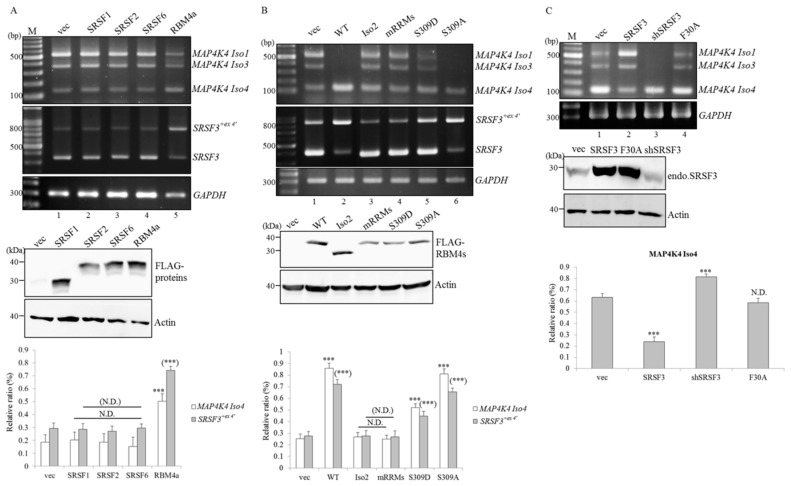
Overexpression of RNA-binding motif protein 4a (RBM4a) specifically reprograms splicing profiles of *mitogen-activated protein kinase kinase kinase kinase 4* (*MAP4K4*) and *serine/arginine-rich splicing factor 3* (*SRSF3*) transcripts. (**A**) Total RNAs and cell extracts were prepared from C3H10T1/2 cells respectively transfected with an expression vector encoding various SRSF family members or RBM4a, followed by culturing in growth medium for 24 h. (**B**) Total RNAs and cell extracts were isolated from C3H10T1/2 cells respectively transfected with an expression vector encoding wild-type (WT) RBM4a or the derived mutants, followed by culturing in growth medium for 24 h. (**C**) Total RNAs and cell lysates were extracted from C3H10T1/2 cells respectively transfected with an SRSF3-targeting vector, or expression vector encoding WT SRSF3 or the derived mutant, followed by culturing in growth medium for 24 h. Splicing profiles of *MAP4K4* and *SRSF3* transcripts were analyzed using RT-PCR assays with specific primer sets as listed in Supplementary Table S1. Immunoblot analyses were performed using specific antibodies against FLAG-tagged proteins, GAPDH, and SRSF3. The bar graph presents relative levels of *MAP4K4* Iso 4 and *SRSF3^+ex4^^′^* transcripts in independent experiments (*n* = 4). Signal densities of the RT-PCR results were analyzed using TotalLab Quant Software, and quantitative results are shown as the mean ± SD (* *p* < 0.05; ** *p* < 0.01; *** *p* < 0.005), N.D., No Difference.

Results of Spearman’s correlation test verified respective coefficients of 0.68 and 0.63 by comparing increases in relative levels of *MAP4K4* Iso 4 and *SRSF3^+ex4′^* transcripts and the upregulated RBM4a protein throughout the development of WT BATs (*n* = 4; *p* = 0.0207 and 0.0334, respectively). An in vitro cell model was next applied to validate impacts of RBM4a on splicing profiles of endogenous *MAP4K4* and *SRSF3*. Results of RT-PCR assays showed that overexpression of RBM4a led to increases in the relative levels of *MAP4K4* Iso 4 and *SRSF3^+ex4′^* (Figure 2A, lane 5) compared to empty-vector transfectants (Figure 2A, lane 1). Splicing profiles of *MAP4K4* and *SRSF3* showed no responses to the overexpression of other SRSF family members (Figure 2A, lanes 2–4). The presence of overexpressed RBM4a Iso 2 and the engineered RBM4a mutant containing four amino acid substitutions (Y37A, F39A, Y113A, and F115A) within two RNA recognition motifs exerted no effect on altering splicing profiles of *MAP4K4* and *SRSF3* transcripts (Figure 2B, lanes 3 and 4) compared to that of empty-vector transfectants (Figure 2B, lane 1). The serine-to-aspartate (S309D) substitution partially interfered with the influence of derived RBM4a mutant on yields of *MAP4K4* Iso 4 and *SRSF3^+ex4′^* transcripts (Figure 2B, lane 5), whereas the derived RBM4a S309A mutant exhibited similar effects of enhancing levels of *MAP4K4* Iso 4 and *SRSF3^+ex4′^* transcripts (Figure 2B, lane 6) compared to overexpressed RBM4a. In contrast, RT-PCR results showed that overexpression of SRSF3 resulted in a decrease in the relative level of *MAP4K4* Iso 4 (Figure 2C, lane 2, PII = 23.96%), whereas ablation of endogenous SRSF3 had a similar effect as that of overexpressed RBM4a on enhancing yields of *MAP4K4* Iso 4 (Figure 2C, lane 3, PII = 81.47%) compared to empty-vector transfectants (Figure 2C, lane 1, PII = 63.27%). Nevertheless, the presence of the derived SRSF3 mutant harboring amino acid substitution (F30A) within the RNA recognition motif exhibited no effect on splicing profiles of *MAP4K4* transcripts (Figure 2C, lane 4, PII = 58.46%). These results suggested that RBM4a, SRSF3, and MAP4K4 constituted an alternative splicing circuit during brown adipogenesis.

### 2.4. RBM4a Modulates the Alternative Splicing of SRSF3 Transcripts in a Sequence-Specific Manner

**Figure 3:**
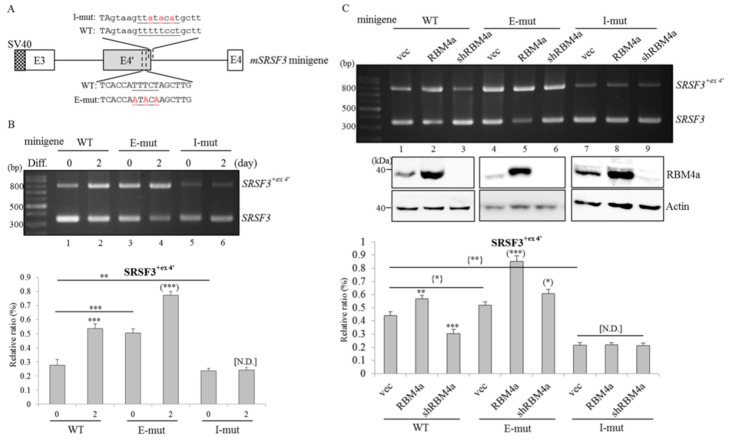
Overexpression of RNA-binding motif protein 4a (RBM4a) leads to an upregulated level of *serine/arginine-rich splicing factor 3* (*SRSF3*)*^+ex4^^′^* transcripts in a intronic CU element-dependent manner. (**A**) The diagram presents the sequence of CU elements (underlined) within mouse *SRSF3* exon 4′ and the downstream intron. (**B**) C3H10T1/2 cells were respectively transfected with the wild-type (WT) *SRSF3* reporter and derived mutants, followed by culturing in growth (0 day) or differentiating (2 day) medium (*n* = 4). (**C**) An empty vector, or RBM4a-expressing vector, or RBM4a-targeting vector were respectively co-transfected with the WT *SRSF3* minigene or derived mutants into C3H10T1/2 cells (*n* = 4). Total RNAs and cell extracts were isolated and subjected to RT-PCR and immunoblotting assays using primer sets as listed in Supplementary Table S1 and specific antibodies. The bar graph shows relative levels of *SRSF3^+ex4^^′^* transcripts. Signal densities of the RT-PCR results were quantified using TotalLab Quant Software. Quantitative results are shown as the mean ± SD. Statistical significance was determined using Student’s unpaired *t*-test (* *p* < 0.05; ** *p* < 0.01; *** *p* < 0.005), N.D., No Difference.

The interplay between the heterogeneous nuclear ribonucleoprotein (HnRNP) I (also referred to as PTBP1) and the UUUCU element within *SRSF3* exon 4′ was documented to impair SRSF3-mediated autoregulation [17]. We therefore wondered whether RBM4a exhibited an antagonistic effect on PTBP1-regulated *SRSF3* splicing as demonstrated in previous reports [18]. The reporter containing intact *SRSF3* exon 3, intron 3, and exon 4 elements and nucleotide-substituted mutants were constructed and applied to in vivo splicing assays (Figure 3A). Results of RT-PCR assays showed that an increase in *SRSF3^+ex4′^* transcripts generated from the WT reporter was observed after treatment with the differentiating cocktail compared to undifferentiated cells (Figure 3B, lanes 1 and 2, PII = 27.54% and 53.8%). Nucleotide substitutions within the UUUCU element of *SRSF3* exon 4′ led to elevation of *SRSF3^+ex4′^* transcripts in transfected cells cultured in growth and differentiating media (Figure 3B, lanes 3 and 4, PII = 50.48% and 77.32%), whereas nucleotide substitutions within the intronic CU element diminished utilization of *SRSF3* exon 4′ in both undifferentiated and differentiated cells (Figure 3B, lanes 5 and 6, PII = 23.67% and 24.24%) compared to those of the WT reporter. Moreover, overexpression of RBM4a mediated increases in relative levels of *SRSF3^+ex4′^* transcripts generated from the WT reporter and exonic mutant (Figure 3C, lanes 2 and 5, PII = 56.8% and 85.27%) compared to empty-vector transfectants (Figure 3C, lanes 1 and 4, PII = 44.19% and 52.02%). In contrast, reduced yields of *SRSF3^+e 4′^* transcripts generated from the WT reporter and exonic mutant were noted after depletion of endogenous RBM4a (Figure 3C, lanes 3 and 6, PII = 30.42% and 43.47%). Nevertheless, splicing profiles of the *SRSF3* intronic mutant showed no response to altered levels of RBM4a compared to empty-vector transfectants (Figure 3C, lanes 7–9). These results suggested that RBM4a mainly modulated utilization of *SRSF3* exon 4′ through the intronic CU element which potentially functions as a splicing enhancer.

### 2.5. RBM4a and SRSF3 Exert Antagonistic Effects on Utilization of MAP4K4 Exon 17

**Figure 4:**
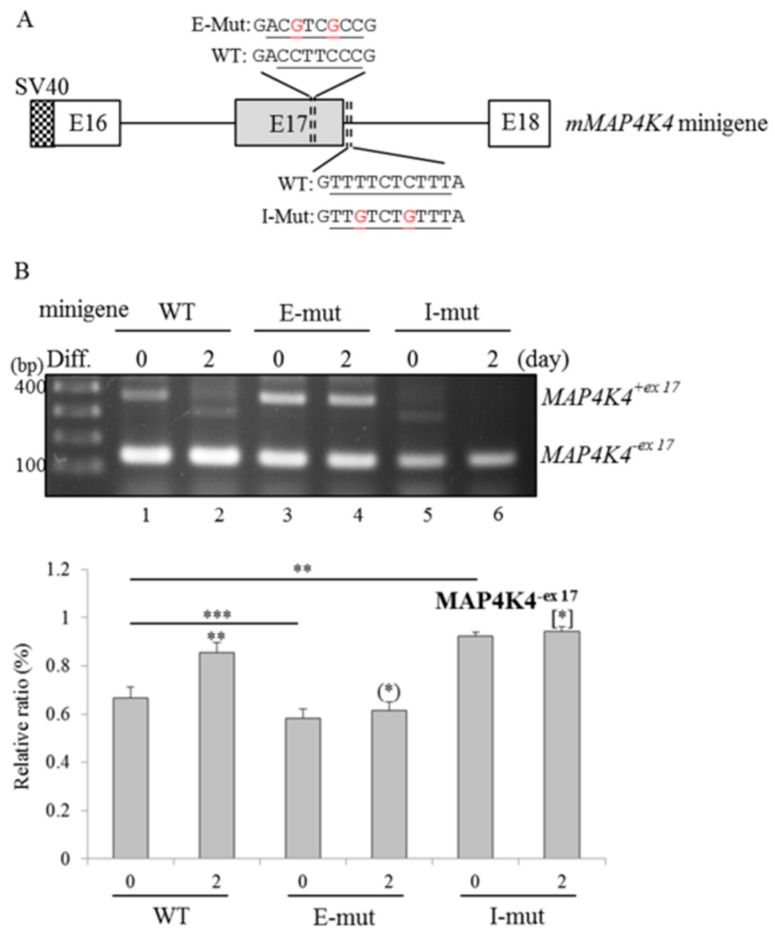

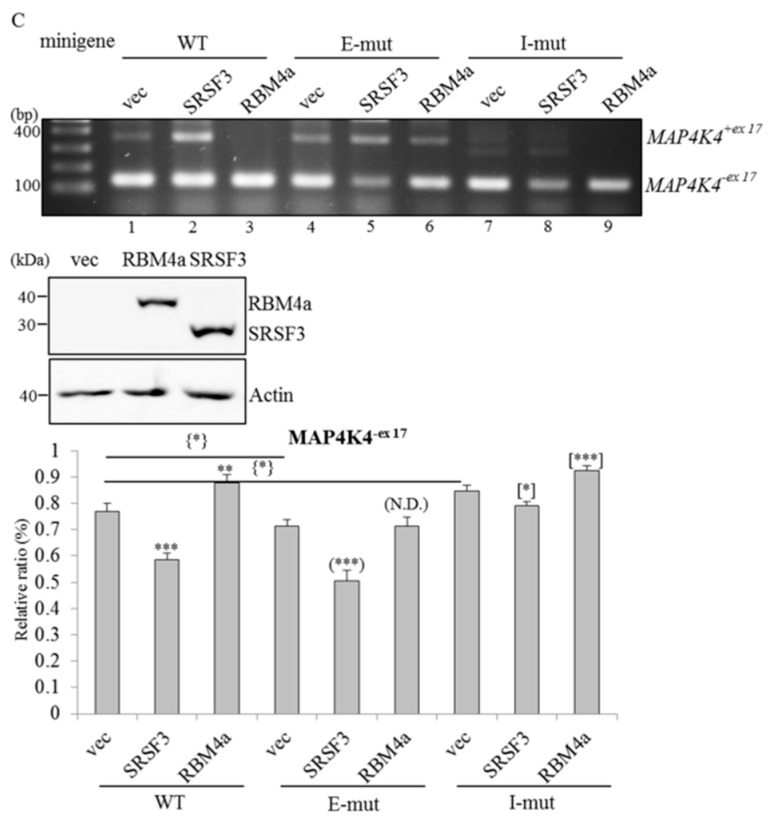
RNA-binding motif protein 4a (RBM4a) and serine/arginine-rich splicing factor 3 (SRSF3) discriminatively modulate the selection of *mitogen-activated protein kinase kinase kinase kinase 4* (*MAP4K4*) exon 17 by interacting with distinct CU elements. (**A**) The scheme shows sequences of CU elements (underlined) within mouse *MAP4K4* exon 17 and the downstream intron. (**B**) The wild-type (WT) *MAP4K4* reporter and derived mutants were respectively transfected into C3H10T1/2 cells, followed by maintenance in growth (0 day) or differentiating (2 days) medium (*n* = 4). (**C**) The WT *MAP4K4* reporter and derived mutants were respectively co-transfected with an expressing vector encoding RBM4a or SRSF3 into C3H10T1/2 cells. Total RNAs and cell extracts were prepared from transfected cells after 24 h and subjected to RT-PCR and immunoblotting assays with primer sets as listed in Supplementary Table S1 and specific antibodies (*n* = 4). The bar graph shows relative levels of *MAP4K4^−ex17^* transcripts. Signal densities of the RT-PCR results were quantified using TotalLab Quant Software. Quantitative results are shown as the mean ± SD. Statistical significance was determined using Student’s unpaired *t*-test (* *p* < 0.05; ** *p* < 0.01; *** *p* < 0.005), N.D., No Difference.

In addition to *MAP4K4* iso 4, inclusion of *MAP4K4* exon 17 was identified in other *MAP4K4* variants. By surveying the genomic sequence of the *MAP4K4* transcript, a high-affinity CU element (CUCUUU) of SRSF3 was noted within *MAP4K4* intron 17 (Figure 4A) [19]. To validate the influence of RBM4a and SRSF3 involved in utilization of *MAP4K4* exon 17, the WT *MAP4K4* minigene and derived mutants were constructed and applied to the following reporter assays (Figure 4A). As shown in Figure 4B, results of RT-PCR analyses showed that treatment with the differentiating cocktail mediated an increase in *MAP4K4^−ex17^* transcripts generated from the WT *MAP4K4* reporter compared to undifferentiated cells (Figure 4B, lanes 1 and 2, PII = 66.82% and 85.38%). Reduced expressions of *MAP4K4^−ex17^* transcripts generated from the exonic mutant were noted in both undifferentiated and differentiated cells (Figure 4B, lanes 3 and 4, PII = 58.18% and 61.55%) compared to those of the WT minigene. In contrast, nucleotide substitutions within the intronic CU element further strengthened the influence of the differentiating cocktail on enhancing relative levels of *MAP4K4^−ex17^* transcripts (Figure 4B, lane 6, PII = 94.17%) compared to that of the WT reporter (Figure 4B, lane 2 PII = 85.38%). Overexpression of RBM4a enhanced yields of *MAP4K4^−ex17^* transcripts generated from the WT *MAP4K4* reporter, whereas a decrease in *MAP4K4^−ex17^* transcripts was identified with the overexpression of SRSF3 (Figure 4C, lanes 2 and 3, PII = 58.47% and 87.66%) compared to the empty-vector transfectant (Figure 4C, lane 1, PII = 77%). In contrast, the splicing profile of the exonic mutant showed no response to the presence of overexpressed RBM4a compared to the empty-vector transfectant (Figure 4C, lanes 4 and 5, PII = 71.26% and 71.38%). Overexpression of SRSF3 continually enhanced yields of *MAP4K4^+ex17^* transcripts generated from the exonic mutant (Figure 4C, lane 6, PII = 50.48%). In contrast, a nucleotide substitution within *MAP4K4* intron 17 lessened the impact of overexpressed SRSF3 on enhancing inclusion of exon 17 compared to that of the empty-vector transfectant (Figure 4C, lanes 7 and 8, PII = 84.77% and 78.96%), whereas overexpressed RBM4a exhibited a repressive effect on the selection of exon 17 (Figure 4C, lane 7, PII = 92.46%). These results suggested that the interplay between RBM4a and the exonic CU element constituted a splicing silencer toward inclusion of *MAP4K4* exon 17. The interaction between SRSF3 and the intronic CU element functioned as a splicing enhancer to maintain the homeostasis of *MAP4K4* variants by antagonizing RBM4a-mediated regulation involved in *MAP4K4* splicing.

### 2.6. MAP4K4 Isoforms Differentially Modulate Activity of the Brown Adipogenesis-Related Signaling Pathway

**Figure 5:**
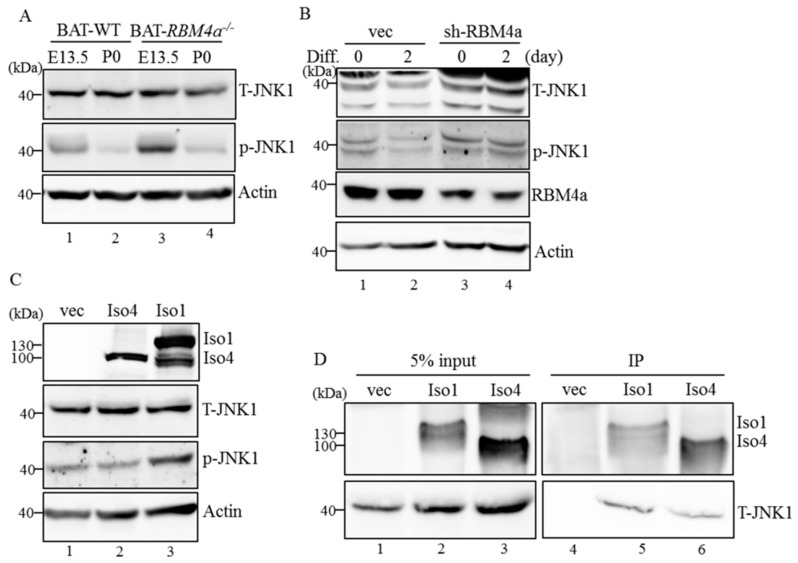
Mitogen-activated protein kinase kinase kinase kinase 4 (MAP4K4) isoforms differentially manipulate activation of c-Jun N-terminal kinase (JNK) signaling throughout brown adipogenesis. (**A**) Cell extracts were prepared from embryonic or postnatal brown adipose tissues (BATs) or (**B**) in vitro cultured cells transfected with an RNA-binding motif protein 4a (RBM4a)-targeting vector, followed by culturing in growth or differentiating medium. (**C**) C3H10T1/2 cells were respectively transfected with an empty vector or expression vectors encoding MAP4K4 isoforms. The cells extracts were isolated after 24 h and subjected to immunoblot assays. (**D**) Cell extracts were isolated from FLAG-tagged MAP4K4 Iso-overexpressing cells, followed by incubation with anti-FLAG M2 agarose. Cell extracts and precipitated complexes were analyzed with immunoblot assays using indicated antibodies (*n* = 4).

Treatment with rapamycin was documented to reduce insulin sensitivity and mitochondrial activity of BAs, which was prohibited in the presence of a JNK inhibitor (19). Moreover, expressions of thermogenic genes by BAs were induced after depletion of MAP4K4 which served as an activator of the JNK signaling pathway [20,21]. Potential impacts of MAP4K4 isoforms on JNK signaling were therefore investigated using an in vitro cell model. Results of an immunoblot assay showed gradual decreases in phosphorylated (p)-JNK-1 throughout the development of BATs (Figure 5A, left, lanes 1 and 2) and differentiation of C3H10T1/2 cells (Figure 5B, lanes 1 and 2), whereas depletion of RBM4a impaired the reduction in p-JNK-1 along with the development of BATs (Figure 5A, lanes 3 and 4) and cultured cells (Figure 5B, lanes 3 and 4). Nevertheless, total JNK expression was not altered by the differentiating condition or RBM4a ablation (Figure 5A,B, T-JNK). Phosphorylation of JNK-1 was induced only in the presence of overexpressed MAP4K4 Iso 1, but not MAP4K4 Iso 4, compared to that of the empty-vector transfectant (Figure 5C, p-JNK, lanes 1–3). Overexpressed MAP4K4 isoforms exhibited no influence on expression of total JNK (Figure 5C, T-JNK) or the loading control. Results of IP assays showed that JNK-1 protein was more associated with FLAG-tagged MAP4K4 Iso1 than with overexpressed MAP4K4 Iso4 in transfected cells (Figure 5D, lanes 5 and 6), which may have subsequently led to the differential phosphorylation of JNK-1 proteins (Figure 5C). Taken together, RBM4a-mediated splicing constituted a regulatory mechanism for manipulating the impact of MAP4K4 on JNK-1 activation.

### 2.7. MAP4K4 Isoforms and SRSF3 Exert Discriminative Effects on Brown Adipogenesis

**Figure 6:**
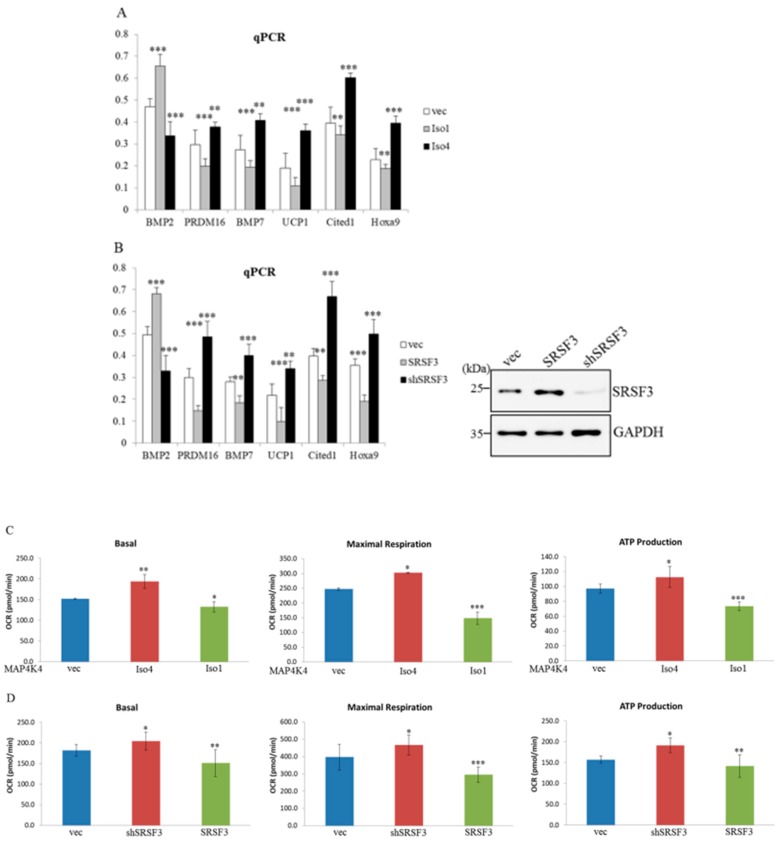
Mitogen-activated protein kinase kinase kinase kinase 4 (MAP4K4) isoforms and serine/arginine-rich splicing factor 3 (SRSF3) exhibit differential impacts on brown adipogenic gene expressions and metabolic signatures. (**A**,**B**) C3H10T1/2 cells were respectively transfected with an empty vector, expression vectors encoding MAP4K4 isoforms, or SRSF3-overexpressing or SRSF3-targeting vectors (*n* = 4). Total RNAs were prepared from transfected cells and subjected to qPCR assays with specific primer sets as listed in Supplementary Table S2. The bar graph shows relative levels of adipogenic genes normalized with the levels of *Gapdh* transcripts. (**C**,**D**) C3H10T1/2 cells were respectively transfected with an empty vector, expressing vectors encoding MAP4K4 isoforms, or SRSF3-overexpressing, or SRSF3-targeting vector, followed by culturing in growth medium for 24 h and then were subjected to bioenergetic analyses. The bar graph shows mean values of the basal and maximal oxygen consumption rates, and ATP production which were measured using a Seahorse XF24 Bioanalyzer (*n* = 4). Quantitative results are shown as the mean ± SD. Statistical significance was determined using Student’s unpaired *t*-test (* *p* < 0.05; ** *p* < 0.01; *** *p* < 0.005).

An in vitro cell model coupled with overexpression of MAP4K4 isoforms or alteration of SRSF3 abundances was next conducted to validate the influence of MAP4K4 isoforms and SRSF3 on the development of brown adipogenesis. Results of qPCR assays indicated that the presence of overexpressed MAP4K4 Iso 4 mediated an increase in expression of *uncoupling protein 1* (*UCP1*) transcript, that serve as a key factor involved in non-shivering thermogenesis in BAs compared to the empty-vector transfectant (Figure 6A, UCP1). In contrast, overexpression of MAP4K4 Iso1 resulted in reduced level of *UCP1* transcript. An increase in *BMP2* transcripts with concomitant decreases in brown adipocytes-specific (*PRDM 16* and *BMP7*) and beige cell-related factors (*Cited 1* and *Hoxa9*) were noted in the MAP4K4 Iso 1—and SRSF3-overexpressing cells (Figure 6A,B, gray bar) compared to those of the empty-vector transfectants (Figure 6A,B, white bar). Overexpression of MAP4K4 Iso 4 and depletion of SRSF3 reversely manipulated the expression profiles of adipocyte-related transcription factors (Figure 6A,B, black bar). Besides the altered expression profiles of a thermogenic factor, overexpression of MAP4K4 Iso 4 mediated elevated basal and maximal OCRs, and ATP production (Figure 6C, red bar) compared to those of empty-vector transfected C3H10T1/2 cells (Figure 6C, blue bar). Reversely, overexpression of MAP4K4 Iso1 resulted in reduced basal and maximal OCRs, and ATP production (Figure 6C, green bar). SRSF3-knockdown cells also exhibited elevated basal and maximal OCRs, and ATP production (Figure 6D, red bar), whereas the reduced basal and maximal OCRs, and ATP production were monitored in SRSF3-overexpressing cells (Figure 6D, green bar) compared to empty-vector transfectants (Figure 6D, blue bar). These results indicated the repressive influence of MAP4K4 iso 1 and SRSF3 on brown adipogenesis, whereas the presence of MAP4K4 iso 4 enhanced the physiological signatures of brown adipocytes.

## 3. Discussion

AS cascades constitute a common mechanism for determining cell and tissue development. For instance, NMD-coupled autoregulation and cross-regulation of PTBP1 and neuronal PTB (also referred to as PTBP2) led to reprogramming of splicing networks throughout neuronal development [22]. In this report, a postnatal switch of the SRSF3 and RBM4a protein expression reprogrammed splicing profiles of *MAP4K4* transcripts, which lessened the repressive effect of JNK signaling on the development and functioning of BATs.

MAP4K4 contains a serine/threonine kinase domain at the N-terminus and a citron-homology (CNH) domain at the C-terminus which participate in protein interactions [23]. The CNH and kinase domains of the MAP4K4 protein were demonstrated to be essential for activation of downstream signaling, including the JNK pathway [24]. Functional MAP4K4 isoforms were previously identified to contain a distinct intermediate region and a regulatory region at the C-terminus which are encoded by alternatively spliced transcripts [24,25]. Despite this, the influences of distinct *MAP4K4* isoforms on carcinogenesis or adipogenesis are as yet uncharacterized. Repressive effects of MAP4K4 on insulin sensitivity, oxidative metabolism, and adipogenesis were revealed using RNA interference (RNAi)-based screening [26]. MAP4K4 was next documented to abolish adipogenesis by downregulating the expression of peroxisome proliferator-activated receptor (PPAR) gamma protein through various signaling pathway, including Wnt, Tumor necrosis factor alpha, and c-JNK pathway [27,28,29]. Although the influence of JNK signaling on adipogenesis was controvertible, treatment with an immunosuppressive agent, such as rapamycin, was recently demonstrated to diminish insulin sensitivity and mitochondrial activity in skeletal muscles and BAs through a MAP4K4-activated JNK pathway [19,27]. Application of a JNK inhibitor inversely lessened the influence of the MAP4K4-mediated pathway on reducing insulin signaling in BATs [19]. In this study, the presence of MAP4K4 Iso4 alone was first reported to enhance the differentiation and metabolism of pre-adipocytes, which may release the repressive effect of MAP4K4 Iso1 throughout brown adipogenesis. These results suggested that the effect of functional protein can be fine-tuned by manipulating the composition of encoded isoforms instead of altering the transcriptional activity. Nevertheless, the influence of *MAP4K4* gene on development of WATs or beige cells was worthy of further investigation.

The SRSF family functions as a major group that orchestrates post-transcriptional regulation to coordinate spatial and temporal gene profiles involved in diverse cellular processes, including differentiation and maturation [28,29]. The AS-coupled NMD mechanism constitutes a common mechanism for auto- and cross-regulation of SRSF family members, such as SRSF3, SRSF6, and other splicing regulators [18,30,31]. The binding tendency of SRSF3 toward the CU element (CUCUUC), which frequently functions as an exonic splicing silencer, was identified using cross-linked IP-coupled sequencing and subsequent functional assays [32,33]. The interplay between SRSF3 and a corresponding element within the 3′ region of its own exon 4′ led to an increase in NMD-targeted *SRSF3* transcripts, subsequently maintaining the homeostasis of SRSF3 expression [17]. Upregulation of hnRNP proteins was documented to impair the autoregulation of SRSF3 splicing and subsequently resulted in imbalanced expression of SRSF3 in various cancers [17]. The simultaneous interplay between RBM4a and the CU element within the *SRSF3* exon 4′ and downstream intron constituted another mechanism for maintaining the homeostasis of SRSF3 during BAT development. Nevertheless, the regulation of SRSF3 splicing throughout BAT development and the repressive effect of SRSF3 on metabolic activity of BAs was first reported in this study. Although the reduced mass of BATs was previously noted in the *SRSF3*-knockout mice [34], overexpression of SRSF3 was demonstrated to enhance the relative levels of *PKM2* transcripts which were predominantly transcribed in WAs [35]. These controversial results suggested that proper expressions of SRSF3 might play a potential role in the development of BAT or other adipose tissues, which is worthy of further investigation.

A gradual increase in RBM4a was previously demonstrated to reprogram splicing profiles of transcription and splicing regulators throughout brown adipogenesis. In this study, a novel splicing network composed of *RBM4a*, *SRSF3*, and *MAP4K4* was identified in BAs using a transcriptome analysis, and subsequently manipulating the effect of downstream JNK signaling which contributes to insulin resistance and reduced metabolism. Therefore, the impacts and regulatory mechanism of a brown adipogenic splicing network are worthy of further investigation, which could function as a therapeutic target for obesity-related diseases.

## 4. Materials and Methods 

### 4.1. Ethics Statement for Animal Research

Animal experiments were approved by the Institutional Animal Care and Use Committee of Taipei Medical University (no. LAC-2016-0351) and were conducted according to relevant guidelines to minimize animal suffering. The creation of *RBM4a^−/−^* mice and isolation of interscapular BATs at distinct stages were described in previous studies [14,16].

### 4.2. Transcriptome Analyses

Transcriptome assays of mice BATs were performed as in previous studies [15]. Briefly, total RNAs were purified using a PureLink RNA mini kit (Invitrogen, Carlsbad, CA, USA) according to the manufacturer’s protocol. Total RNAs (8 μg) with a high integrity number (RIN > 8.0) were used for library construction with the NEBNext Ultra RNA Library Prep Kit (NEB, Ipswich, MA, USA) according to the manufacturer’s instructions. Paired-end reads (150 bp) were amplified on an Illumina Hi-Seq 4000 platform with the prepared libraries. Preliminary reads were trimmed, filtered, and aligned to the mouse reference genome (GRCm37) using the Tophat v2.0.9 program. Aligned reads were applied to transcriptome assemblies using the Cufflink program. Transcriptome assemblies generated from individual samples were merged using the Cuffmerge utility to estimate transcript levels in each condition. Expression levels and the statistical significance of the merged assemblies were examined using a Cuffdiff and CLC genomic workbench analysis.

### 4.3. Cell Culture and In Vitro Differentiation

Mouse C3H10T1/2 fibroblast cells were cultured in Dulbecco’s modified Eagle medium (DMEM; Invitrogen) supplemented with 10% fetal bovine serum (FBS; Invitrogen). In vitro differentiation was conducted by shifting C3H10T1/2 cells to induction medium supplemented with 20% FBS, 0.5 mM IBMX, 12.7 μM dexamethasone, and 10 µg/mL insulin. The induction medium was replaced with differentiation medium (DM) supplemented with 10% FBS and 10 μg/mL insulin after 48 h and replenished every 2 days.

### 4.4. Plasmid Construction

Coding regions of human *MAP4K4* and *SRSF3* were amplified using a polymerase chain reaction (PCR) with a mice fetal complementary (c)DNA library as the template. The PCR product was digested with *Hind* III*/EcoR* I and *EcoR* I*/EcoR* V restriction enzymes and inserted into the p3XFLAG-CMV14 vector (Sigma, St. Louis, MO, USA). The mouse genomic element containing *SRSF3* exon 3, intron 3, and exon 4 was PCR-amplified with genomic DNA prepared from C3H10T1/2 cells as the template. The PCR product was digested with *EcoR* V and *Sac* I restriction enzymes, and then the insert was placed into the pUC19 vector (NEB). The *MAP4K4* minigene was constructed by inserting mouse *MAP4K4* genomic fragments containing exon 15, exon16, exon 18, and the complete introns into the pUC19 vector (NEB). PCR-amplified fragments were digested with *EcoR* I and *Hind* III restriction enzymes prior to DNA ligation. Derived mutants of the expressing vectors or minigene reporters harboring substituted nucleotides were constructed using the QuikChange site-directed mutagenesis system (Stratagene, Amsterdam, The Netherlands). The vector-based short hairpin RNA targeting mouse *SRSF3* was purchased from the RNAi core facility at Academia Sinica (Taipei, Taiwan).

### 4.5. Transient Transfection, Reverse-Transcription (RT)-PCR, and Quantitative (q)PCR Analyses

Cultured C3H10T1/2 cells at ~60% confluence were transfected with the indicated plasmid using Lipofectamine 3000 according to the manufacturer’s protocol (Invitrogen). Total RNAs and cell extracts were isolated using a PureLink RNA mini kit (Invitrogen) at 24 h post-transfection. Total RNA (1 µg) was reverse-transcribed using SuperScriptase III (Invitrogen) in a 10-μL reaction and then subjected to PCR assays with specific primer sets (Supplementary Table S1). Levels of *GAPDH* transcripts served as a loading reference. The qPCR assay was performed using SYBR green fluorescent dye and gene-specific primer sets (Supplementary Table S2) on an ABI One Step™ PCR machine (Applied Biosystems, Foster City, CA, USA). The relative messenger (m)RNA level was quantitated by the ΔΔ−Ct method, and normalized to levels of *GAPDH* transcripts.

### 4.6. Co-Immunoprecipitation (Co-IP) and Immunoblot Assays

An expressing vector encoding the FLAG-tagged MAP4K4 iso1 and iso4 was transfected into C3H10T1/2 cells using Lipofectamine 3000 (Invitrogen). Transfected cells were lysed in RIPA solution containing 10 mM sodium phosphate (pH 7.2), 150 mM sodium chloride, 2 mM EDTA, and 1% Nonidet P-40 at 24 h post-transfection. The FLAG-tagged MAP4K4 isoform and associated protein were immunoprecipitated using anti-FLAG M2 agarose (Sigma) and washed with RIPA buffer containing 0.1% Nonidet P-40. To extract the precipitated complex, the washed beads were incubated at 95 °C for 15 min and removed by centrifugation. The immunoblot analysis was conducted using an enhanced chemiluminescence (ECL) system (Millipore, Billerica, MA, USA), and results were monitored using the LAS-4000 imaging system (Fujifilm, Tokyo, Japan). Primary antibodies used in this study included polyclonal anti-RBM4a (dilution 1:1000; Santa Cruz Biotechnology, Santa Cruz, CA, USA), polyclonal anti-GAPDH (dilution 1:2000; MDBio, Taipei, Taiwan), polyclonal anti-phosphorylated JNK (dilution 1:1000; R&D Systems, Minneapolis, MN, USA), monoclonal anti-SRSF3 (dilution 1:2000; Abnova, Taipei, Taiwan), monoclonal anti-actin (dilution 1:4000; Millipore), monoclonal anti-JNK (dilution 1:1000; R&D Systems), and monoclonal anti-MAP4K4 (dilution 1:500; Cell Signaling Technology, Beverly, MA, USA) Signal intensities were evaluated using TotalLab Quant Software.

### 4.7. Mitochondrial Respiration Assay

The oxygen consumption rate of cultured C3H10T1/2 cells was measured using a Seahorse XF24 bioanalyzer (Seahorse Bioscience, Billerica, MA, USA). In brief, C3H10T1/2 cells (2 × 10^4^) were seeded in wells of Seahorse XF24 plates with 250 µL of DMEM overnight. Prior to the measurement, cells were washed with unbuffered medium and immersed in 675 µL of unbuffered medium without CO_2_ for 1 h. The oxygen consumption rate (OCR) was assessed in 8-min cycles as recommended by Seahorse Bioscience. Basal and maximal OCRs, and the spare respiratory capacity were recorded following injection of complex-specific substrates, including FCCP (2 μM), rotenone (2 μM), and oligomycin (2.5 µg/mL).

### 4.8. Statistical Analyses

An analysis of variance (ANOVA) and Student’s *t*-tests were performed to determine the significance of the experimental results. *p* < 0.05 was considered statistically significant. 

## Figures and Tables

**Table:** 

(A)								
Sample	Symbol	Accession No.	Locus	Length	Coverage	FPKM	*p*-Value	Percentage Isoform Index (NM_001177995)
WT BAT (E13.5)	PRDM16	NM_001177995	chr4:154316124-154636873	8433	2.3435	1.95142	0.0231	35.18%
	PRDM16	NM_027504	chr4:154316124-154636873	8605	3.4659	3.59551	0.0347	
*RBM4a^−/−^* BAT (E13.5)	PRDM16	NM_001177995	chr4:154316124-154636873	8433	0	1.69 × 10^−5^	0.0219	<1%
	PRDM16	NM_027504	chr4:154316124-154636873	8605	4.35909	4.12531	0.0147	
WT BAT (P0)	PRDM16	NM_001177995	chr4:154316124-154636873	8433	4.112475	2.04078	0.0341	>99%
	PRDM16	NM_027504	chr4:154316124-154636873	8605	0	2.71 × 10^−5^	0.0157	
*RBM4a^−/−^* BAT (P0)	PRDM16	NM_001177995	chr4:154316124-154636873	8433	2.102418	1.65639	0.0267	77.24%
	PRDM16	NM_027504	chr4:154316124-154636873	8605	1.112425	0.487891	0.0271	

$\mathrm{Percentage}\;\mathrm{Isoform}\;\mathrm{Index}=\frac{\mathrm{Isoform}\;A}{\sum \mathrm{Isoform}\;A+B}\times 100\%$.

**Table:** 

(B)								
Gene	Locus	Sample-1	Sample-2	FPKM-1	FPKM-2	Log 2 (Fold Change)	*p*-Value	*q*-Value
PRDM16 (E13.5)	chr4:154316124-154636873	BAT-WT	BAT- RBM4a^−/−^	8.21412	6.21324	0.403	0.0397	0.0455
PRDM16 (P0)	chr4:154316124-154636873	BAT-WT	BAT- RBM4a^−/−^	12.0365	7.54704	0.674	0.0241	0.0361
				*p* = 0.0207	*p* > 0.05			

## Supplementary Materials

Supplementary materials can be found at http://www.mdpi.com/1422-0067/19/9/2646/s1.
