# Supplementary material for: RBM4a-SRSF3-MAP4K4 Splicing Cascade Constitutes a Molecular Mechanism for Regulating Brown Adipogenesis
Source: Int J Mol Sci. 2018 Sep 6;19(9):2646. doi: 10.3390/ijms19092646 (PMC6163301; doi:10.3390/ijms19092646)
Supplement: Supplementary file 1 [file ijms-19-02646-s001.zip › ijms-347217-SI.pdf]

Int. J. Mol. Sci. 2018, 19, 2646; doi:10.3390/ijms19092646 www.mdpi.com/journal/ijms

Supplementary Materials

RBM4a-SRSF3-MAP4K4 Splicing Cascade Constitutes a Molecular Mechanism for Regulating Brown Adipogenesis

Hui-Yu Peng [1], Yu-Chih Liang [1,2], Tse-Hua Tan [3], Huai-Chia Chuang [3], Ying-Ju Lin [4] and Jung-Chun Lin [1,2,*]

1 School of Medical Laboratory Science and Biotechnology, College of Medical Science and Technology, Taipei Medical University, Taipei 110, Taiwan; m609104003@tmu.edu.tw (H.-Y.P.); ycliang@tmu.edu.tw (Y-C. L.)
2 Ph.D. Program in Medicine Biotechnology, College of Medical Science and Technology, Taipei Medical University, Taipei 110, Taiwan
3 Immunology Research Center, National Health Research Institutes, Zhunan 35053, Taiwan; ttan@nhri.org.tw (T.-H.T.); cinth@nhri.org.tw (H.-C.C.)
4 School of Chinese Medicine, China Medical University, Taichung 40402, Taiwan; yjlin@mail.cmu.edu.tw
* Correspondence: lin2511@tmu.edu.tw; Tel.: +886-2-2736-1661 (ext. 3330)

Supplemental Table S1. PCR Primers.

Target	Forward	Reverse
MAP4K4-AS	agagcccaagcctcactatgac	tctgtcctgcttggctattct
MAP4K4-cDNA	atcaagcttatggcgaacgactctcccgcg	atcgaattcccagctcagaagagaagtcct
SRSF3-AS	gtcccttggattgtaaggtt	aaaagcttctccttcttggg
SRSF3-cDNA	atcgaattcatgcatcgtgattcctgtc	atcgatatctttcctttcatttgacctag
SRSF3(F30A) mutant	acggaattggaacgggctgctggctactatggaccactc	gagtggtccatagtagccagcagcccgttccaattccgt
MAP4K4-minigene	atcgaattccacgaccacaggaggccgcac	atcaagcttctggtggaatttctctgtcc
MAP4K4-minigene (E-mut)	ctcaggacccatgtccacgttgccgcagtgaggggctca	tgagcccctcactgcggcaacgtggacatgggtcctgag
MAP4K4-minigene (I-mut)	cagacagacgcgctgttgtctgtttatttaaacttactgt	acagtaagtttaaataaacagacaacagcgcgtctgtctg
SRSF3-minigene	atcgatatcaacactatgtggctgccgtg	atcgagctcctgctccggctgcgagagaag
SRSF3-minigene (E-mut)	ccggcagcctcacctcaccaatacaagcttgttgaaa cccaaaa	ttttgggtttcttcttgcttgtattggtgaggtgaggctgccgg
SRSF3-minigene (I-mut)	acccaaaactagtaagttatacatgcttatacagtttact	agtaaactgtataagcatgtataacttactagttttggt
CMV	tgggaggtctatataagcaga	
GAPDH	cggagtcaacggatttggtcgtatg	agccttctccatggtggtgaagac

Supplemental Table S2. qPCR Primers.

Target	Forward	Reverse
PRDM16	gacattccaatcccaccaga	cacctctgtatccgtcagca
BMP2	ccaagagacatgtgaggatt	ttagtggagttcaggtggtc
BMP7	gaaaacagcagcagtgacca	ggtggcgttcatgtaggagt
UCP1	tacacggggacctacaatgct	ggtcactggaagatatggc
Cited1	cggggtcaccgcaaatgga	ctcatccaccgggtcagaa
Hoxa9	cagtgtatcatcaccaccacca	gaggagaaccacaagcatagt
Gapdh	cggagtcaacggatttggtcgtatg	agccttctccatggtggtgaagac
